# Diagnosis and Antiviral Intervention Strategies for Mitigating an Influenza Epidemic

**DOI:** 10.1371/journal.pone.0014505

**Published:** 2011-02-04

**Authors:** Robert Moss, James M. McCaw, Jodie McVernon

**Affiliations:** Vaccine and Immunisation Research Group, Murdoch Childrens Research Institute and Melbourne School of Population Health, The University of Melbourne, Parkville, Australia; Centers for Disease Control and Prevention, United States of America

## Abstract

**Background:**

Many countries have amassed antiviral stockpiles for pandemic preparedness. Despite extensive trial data and modelling studies, it remains unclear how to make optimal use of antiviral stockpiles within the constraints of healthcare infrastructure. Modelling studies informed recommendations for liberal antiviral distribution in the pandemic phase, primarily to prevent infection, but failed to account for logistical constraints clearly evident during the 2009 H1N1 outbreaks.

Here we identify optimal delivery strategies for antiviral interventions accounting for logistical constraints, and so determine how to improve a strategy's impact.

**Methods and Findings:**

We extend an existing SEIR model to incorporate finite diagnostic and antiviral distribution capacities. We evaluate the impact of using different diagnostic strategies to decide to whom antivirals are delivered. We then determine what additional capacity is required to achieve optimal impact. We identify the importance of sensitive and specific case ascertainment in the early phase of a pandemic response, when the proportion of false-positive presentations may be high. Once a substantial percentage of ILI presentations are caused by the pandemic strain, identification of cases for treatment on syndromic grounds alone results in a greater potential impact than a laboratory-dependent strategy. Our findings reinforce the need for a decentralised system capable of providing timely prophylaxis.

**Conclusions:**

We address specific real-world issues that must be considered in order to improve pandemic preparedness policy in a practical and methodologically sound way. Provision of antivirals on the scale proposed for an effective response is infeasible using traditional public health outbreak management and contact tracing approaches. The results indicate to change the transmission dynamics of an influenza epidemic with an antiviral intervention, a decentralised system is required for contact identification and prophylaxis delivery, utilising a range of existing services and infrastructure in a “whole of society” response.

## Introduction

Governments and public health agencies around the world extensively revised influenza pandemic preparedness strategies in the early 21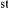 century, primarily in response to the H5N1 avian influenza epizootic and its attendant risks to humans [1], [2]. With many other developed countries, Australia amassed large stockpiles of the neuraminidase inhibitors oseltamivir and zanamivir in anticipation of such a public health emergency [3]. The AHMPPI placed considerable emphasis on constraining spread of the virus through early case detection and isolation, with quarantine and provision of chemoprophylaxis to close contacts [4]. Two phases with differential intensity of case-finding termed “Contain” and “Sustain” were described in which antivirals were employed as a key strategy to limit the growth rate of the epidemic, in order to “buy time” for roll-out of strain-specific vaccine (“Control” phase) [4]. The target clinical attack rate by which successful mitigation was defined in planning scenarios was 10% or less [4]. [5].

Given the very recent availability of NAIs for such widespread use [6], [7], there was no relevant field experience to inform optimal deployment. Mathematical models of population transmission were used to infer likely effects on epidemic dynamics [8]–[14], using data from human and animal studies of experimental infection [15], [16] and efficacy trials conducted within the household unit [17], [18]. Model findings informed recommendations for liberal antiviral distribution early in pandemic responses [19], [20], primarily for prevention of infection [8], [21].

These strategies were put to the test in the influenza A (H1N1) 2009 pandemic. In Australia, the implementation of the “Contain” strategy lasted for several weeks in some Australian states, prior to switching to a more proportionate “Protect” phase given the generally mild nature of observed disease. In the planning phase, sufficient stockpiling and distribution of resources, along with rapid and clear two-way communications were identified as critical determinants of success both within Australia [22], [23] and internationally [19], [20]. Further issues identified in the media and medical press by critics of the pandemic response included excessive administrative burden on general practices (GPs), delays in receiving test results, centralised bottlenecks, a lack of clear communication, updates to the AHMPPI that some considered “not entirely workable” and that were applied inconsistently, inadequately detailed planning and other real-world complexities [21], [24]–[26].

In particular, delays in diagnosis and antiviral distribution reduce the impact of an antiviral intervention. In comparison to laboratory-based molecular diagnosis tests such as polymerase chain reaction (PCR), rapid point-of-care tests (POCTs) may help reduce these delays by providing an immediate (albeit less sensitive) diagnosis option. The use of these POCT s to help contain an influenza outbreak has been studied in the context of the 2008 World Youth Day [27], [28], where PCRtests did not demonstrate a higher utility when turnaround times were included [28]. Depending on the logistics of a pandemic response, POCT s may prove a more effective diagnostic tool for antiviral distribution than PCRtests.

In addition to the benefits of timely diagnosis, timely surveillance data is critical for appropriately adjusting the healthcare response [29]; widespread use of POCT s could reduce the diagnostic load on laboratories and improve the turnaround of surveillance reports. For example, the Victorian experience of the 2009 epidemic suggests that the influenza circulation was similar to moderate seasonal influenza activity at most [3], [30], but the high workload prevented subtyping of all specimens [31] and laboratories ultimately limited test capacity to high-risk patients [32]. The mild nature of the 2009 pandemic also served to confound the planned interventions due to a low proportion of pandemic infections presenting to healthcare facilities. Given the limits on antiviral distribution and other logistical constraints that were identified in 2009 pandemic responses world-wide [33], the impact of an antiviral intervention strategy on influenza transmission in a future pandemic remains uncertain and requires further investigation.

We identify the optimal diagnostic strategies for antiviral distribution within the logistical constraints of the health services sector observed in the Australian response to the Influenza A H1N1 2009 pandemic, which are likely to be similar (within an order of magnitude) in other developed countries. We then evaluate the relative benefits of investing additional resources in either laboratory or drug distribution capability for intervention effectiveness.

We have extended an existing Susceptible Exposed Infectious Recovered (SEIR) model [9], [10] to account for presentations at multiple locations (hospitals, GP s and flu clinics), the diagnosis and treatment strategies available at each location, and the finite diagnostic and antiviral distribution capacities of the pandemic response. We show the optimal diagnostic strategy for targeting antiviral distribution is to use PCRtests until lab diagnostic capacity is exceeded, and then use syndromic diagnosis from this point. Increased antiviral distribution capacity is shown to greatly improve strategy impact, while increased lab diagnostic capacity is shown to have negligible effect on impact. This is at odds with widespread recommendations to greatly increase lab diagnostic capacity to improve pandemic responses [19], [33].

## Methods

The deterministic model presented here is based on an existing SEIR model that captures disease status and contact status as separate states [9], [10]; full details are given in Supplementary Material S1, S1. Novel features of this extended model include using case severity to determine the likely location of presentation—with implications for diagnostic facilities and treatment—and to predict the effects of having limited diagnostic and antiviral distribution capacities. With this model, different diagnostic strategies were evaluated for their ability to identify sufficient pandemic presentations for antiviral interventions in order to successfully mitigate an influenza epidemic. Antiviral drugs were deployed from a finite stockpile, similar in size to that existing in Australia prior to the 2009 winter [8].

To account for model sensitivity to individual parameters, Latin hypercube sampling (LHS) was used to take many representative samples of the model dynamics across the parameter space. By using this approach we obtained results for thousands of simulated epidemics, and so statistical analyses were used to understand the model behaviour.

The extensions to the original SEIR model are now presented in detail, followed by a description of the epidemic scenarios that were considered and an overview of the analysis techniques that were applied.

### Case severity and presentation

**Figure 1:**
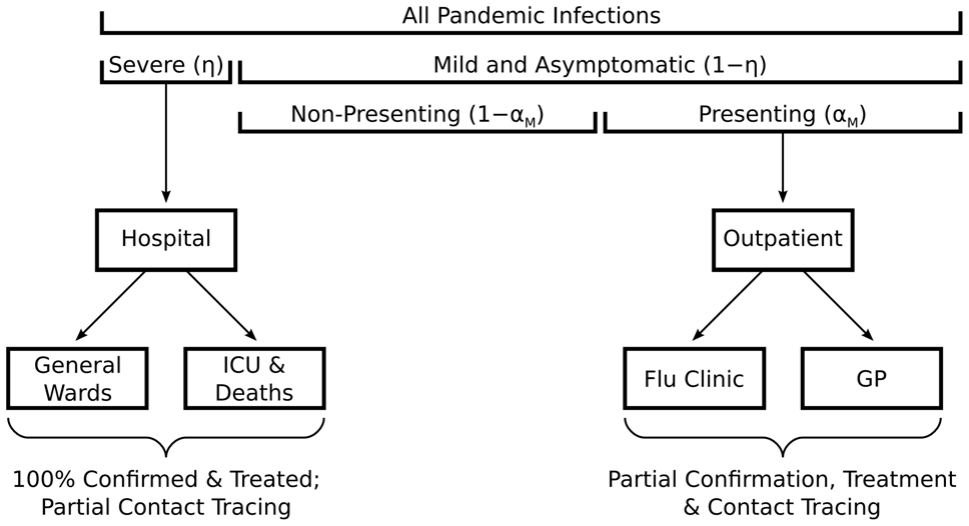
The severity and location of pandemic presentations. Of all pandemic infections (calculated in the model through the SEIR dynamics) a proportion 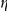 are severe. Of the remainder, 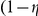), a proportion 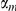 present to outpatient facilities. All severe cases present to hospitals for treatment; a fraction of these cases are admitted to intensive care units (ICUs). Mild cases present to general practices (GPs) and flu clinics for treatment and contact tracing. Effectiveness of antiviral interventions on the different groups accounts for setting-specific losses due to late presentation, testing delays and personnel constraints.

Our assumptions about case severity and presentation are depicted in Figure 1. We assume that all severe cases present for diagnosis at hospitals, and the proportion of pandemic infections that are severe (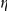) is assumed to be between 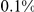 and 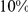. For simplicity, we assume that all flu-like hospital presentations receive diagnosis and treatment without delay, and that the diagnosis is 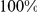 sensitive and specific in this setting. These simplifying assumptions do not compromise the validity of the model, as the treatment of severe cases (whether effective or not) has little impact on the community transmission of pandemic influenza, given that severe cases are only a small proportion of the total case load and that treatment does not significantly reduce infectiousness [18], [34].

Of the remaining cases (mild and asymptomatic), we assume that a fraction 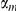 present to outpatient facilities; we understand two models— GP s and flu clinics—with different diagnosis strategies. We assume that outpatient consultation capacity is sufficient for all flu-like presentations to receive timely consultation, and that the proportion of mild cases that present is influenced by the prevalence of severe cases (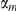 varies from 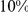 to 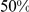 in proportion to 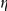). Mild cases are the key to controlling community transmission, and unlike severe cases there is incomplete ascertainment due to limited presentation levels with additional challenges for disease control arising from the imperfect timeliness and precision of diagnosis. Moreover, such cases may not present in a timely way due either to delayed health-care seeking or service availability constraints. During the 2009 H1N1 pandemic response in Australia, individuals presenting beyond the 48-hour window of established antiviral efficacy were not offered antiviral treatment. While such individuals are not explicitly considered within the model, they are implicitly subsumed into the non-presenting proportion.

### Outpatient diagnosis strategies for antiviral deployment

Several diagnostic strategies were evaluated for their ability to target outpatient presentations for antiviral interventions in order to successfully mitigate an influenza epidemic. The time taken to transport samples to external laboratories from GP s is assumed to reduce the effectiveness of any delivered antivirals. For simplification the influenza-like illness (ILI) case definition is assumed to be 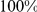 sensitive for pandemic influenza, although this is certainly not true in practice [35], while the specificity is dependant on the proportion of ILI presentations infected with pandemic influenza and varies throughout the epidemic. Of all out-patient presentations, only those matching the ILI case definition are candidates for antiviral treatment, subject to a positive result from the chosen outpatient diagnosis strategy. Post-exposure prophylaxis is also delivered to identified contacts of those who return a positive result.

**Figure 2:**
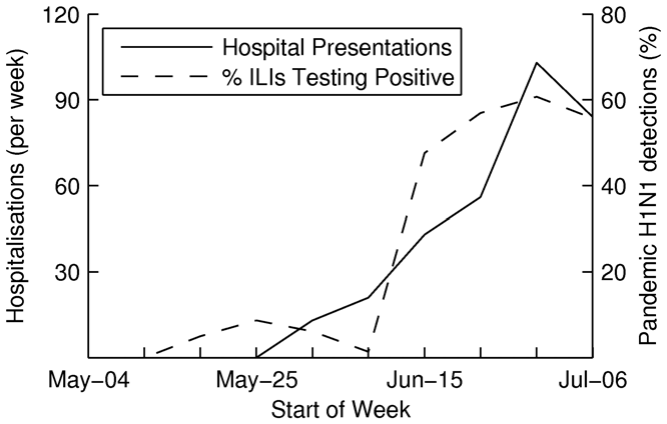
The proportion of ILI cases infected with pandemic influenza increases as the epidemic progresses. This is illustrated by comparing surveillance data for the 2009 epidemic with pandemic influenza hospitalisations (per week) from Victoria, Australia. The proportion of ILI cases infected with pandemic influenza rose from 0% to approximately 60% over a two month period. We use the observed correlation to infer a linear model for the proportion of ILI presentations that are infected with the pandemic strain over time.

Given a finite diagnostic capacity, the number of pandemic presentations that are tested depends on the proportion of ILI presentations that are infected with the pandemic strain. In Figure 2, the number of pandemic influenza hospitalisations (per week) [36] is used to illustrate the timing of the 2009 epidemic in Victoria, Australia, and results from Victorian sentinel surveillance show that as the epidemic progressed, the proportion of outpatient ILI presentations due to pandemic influenza rose from 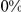 to approximately 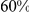 [31], [37]. We fit a linear model to predict the proportion of ILI presentations that are infected with the pandemic strain, which is presented in Supplementary Material S1, S2.1.

We considered five diagnostic strategies, based on some that were deployed in the 2009 pandemic response and others that could conceivably be deployed in the future. The parameters for each diagnostic strategy are listed in Table 1 and we now introduce each in turn.

**Table 1:** Parameters for the diagnostic strategies available at outpatient locations.

	PCR	POCT (lab)	POCT (local)	Syndromic
True Positives	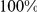	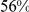	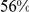	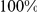
True Negatives	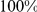	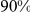	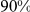	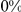
Capacity (diagnoses per day)	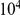	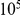	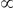	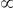
Antiviral Effectiveness ( GP s )	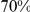	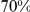	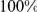	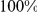

True positives and negatives are the proportion of outpatient presentations meeting the ILI case definition that are correctly identified as being infected and uninfected, respectively, with the pandemic strain. These are not equivalent to sensitivity and specificity values.

#### Molecular diagnosis methods

Molecular diagnosis methods such as PCRtests are resource-intensive and conducted at external laboratories. As when used in the hospital setting, we assume that these “gold standard” methods have perfect sensitivity and specificity. However, when ordered from outpatient facilities, transport delays and turnaround time reduce the effectiveness of delivered antivirals (we assume a reduction of 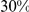) and thus reduce the *effective* sensitivity and specificity of the diagnosis strategy. We estimate a national capacity of 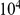 PCRtests per day, based on personal communications with Dominic Dwyer (Westmead Hospital, NSW).

#### Rapid testing

Based on studies of the performance of rapid POCT s [27], [28], we considered two strategies for using these tests to target outpatient presentations for antiviral interventions: on-site testing as performed in the POCT studies, and sending the swabs to external laboratories for analysis. When used in the Australian 2009 pandemic response, the POCT s were sent to external laboratories for analysis because no financial compensation was available for POCT s analysed in near-patient settings; this is reflected in our second POCT strategy. Since POCT s are less time-consuming to analyse than PCRtests, we estimated a diagnostic capacity 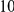 times greater than that for PCRtests. As for molecular diagnosis methods, transport delays and turnaround time reduce antiviral effectiveness by 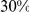.

#### Syndromic diagnosis

In contrast to the molecular and virologic tests introduced above, we also considered targeting antiviral interventions based on the syndromic diagnosis of patients meeting the ILI case definition. Since every ILI presentation is positively diagnosed, all true positives are correctly identified (since we assume that the ILI case definition is 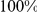 sensitive to pandemic influenza) but every true negative ( i. e. each ILI presentation not infected with the pandemic strain) is erroneously diagnosed.

#### Combined strategy: PCR/syndromic

The final strategy was to use a combination of molecular and syndromic diagnosis to target antiviral interventions. In the early phase of the epidemic, PCRtests are used as the number of cases is low and few ILI presentations are infected with the pandemic strain. Once the PCRdiagnostic capacity is exceeded by the number of ILI presentations, syndromic diagnosis is used as the decision making strategy. The majority of ILI presentations are likely to be infected with the pandemic strain by this stage (demonstrated in Figure 2).

### Antiviral intervention and vaccination

Treatment was delivered to all severe cases and, subject to a maximal delivery rate of 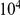 doses per day, treatment was also delivered to all positively diagnosed ILI presentations who had not previously received antivirals for prophylaxis. Prophylaxis, also subject to maximal delivery rate of 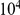 doses per day, was delivered to identified contacts of all severe cases and all positively diagnosed ILI presentations. Studies have shown that people have around 20–30 contacts on average [38], [39], about half of which are readily identifiable (e. g. people they live or work with), and these estimates were used in our model.

The maximal prophylaxis delivery rate of 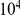 doses per day was based on an aspirational target of providing prophylaxis for contacts of 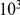 cases per day, on the assumption that around 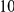 contacts per case receive prophylaxis. The constraint on treatment delivery was also 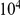 doses per day, but this value is less significant since treatment has minimal effect on transmission in this model.

In addition, a vaccine was introduced to the population at a rate of 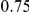 million doses per week (one dose per person), with seroconversion in 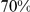 of recipients starting 20 weeks into the epidemic. The vaccine did not provide perfect protection against the pandemic strain but reduced susceptibility by 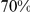, which is similar to existing estimates of vaccine efficacy against susceptibility [40].

### Epidemic scenario

Epidemic scenarios were randomly chosen from predefined distributions for each of the model parameters; these distributions are presented in Supplementary Material S1, S2.2. In brief, we assumed a basic reproductive number of 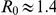 in a fully susceptible population, where treatment had minimal effect on infectiousness and where prophylaxis moderately reduced susceptibility, but breakthrough cases had little reduction in infectiousness. Individuals were assumed to have around 30 contacts during the Contain phase and 20 contacts post-Contain [8], of which half were assumed to be readily identifiable for the purposes of prophylaxis distribution. We chose to evaluate the diagnosis strategies for targeting antiviral interventions in scenarios more severe than the 2009 pandemic, which had an estimated final attack rate of up to 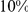 [41] with strong suggestive evidence of prior immunity [42], [43]. We compare our chosen scenarios to the 2009 H1N1 pandemic in Supplementary Material S1, S2.3.

### Model analysis

The model dynamics were analysed using LHS, a biased statistical sampling method for generating plausible collections of parameter values from multidimensional distributions. When taking a number of samples (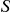) of the model parameters, the range of each parameter is divided into 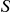 equally probable intervals, and a value is chosen at random from each interval. This ensures that the ensemble of parameter values is representative of the real variability of the parameters, unlike traditional (i. e. “brute force”) random sampling, which provides no such guarantee.

The results presented in this paper were generated by taking 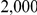 samples of the model parameter space for each value of the *control parameter(s)*; typically the control parameter is the proportion of infections that are severe (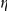). Given the statistical nature of this analysis technique, the impact of a strategy was specified as the percentage of simulations where the final attack rate was less than the 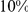 target attack rate specified in the AHMPPI (similar target rates have been used in US studies [44]). In our results, we show that this is a valid measure of impact.

## Results

With the extended SEIR model presented here, several experiments were undertaken to identify: the diagnostic strategies that have the greatest impact; how the impact of using POCT s is affected by their sensitivity; and how the logistical constraints identified in this paper affect the impact of the strategies employed.

### Impact of Diagnosis Strategies

**Figure 3:**
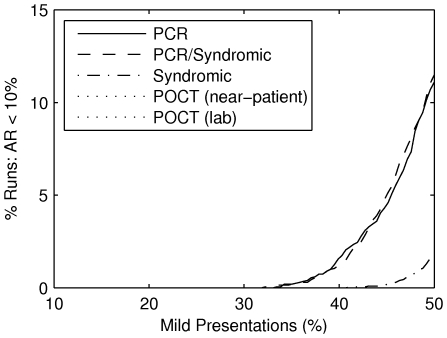
The impact of using each diagnosis strategy, for different presentation rates of mild cases (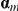). As mild-presentations constitute the bulk of all symptomatic infections, if only a small percentage of them present to outpatient facilities, then an intervention (based on any diagnostic strategy) is unable to control the epidemic. If approximately one in three or more mild cases presents, then there is a non-negligible chance that an epidemic may be controlled given an appropriate diagnostic strategy is employed. The probability of control rises rapidly with the proportion of mild cases that presents. Maximal impact is achieved with the PCR and PCR/Syndromic strategies. A syndromic strategy is less effective. The POCT strategies have negligible impact (the curves lie on the horizontal zero axis).

The impact of any intervention (except population-wide vaccination) is determined in part by the proportion of infected persons that present. Since severity is assumed to be the driver for mild presentations, the impact of each diagnosis strategy increases as the severity (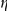) of the epidemic increases. As shown in Figure 3, the strategies with the greatest impact are PCRand PCR/syndromic, followed by syndromic diagnosis, while the two POCT strategies have no impact.

**Figure 4:**
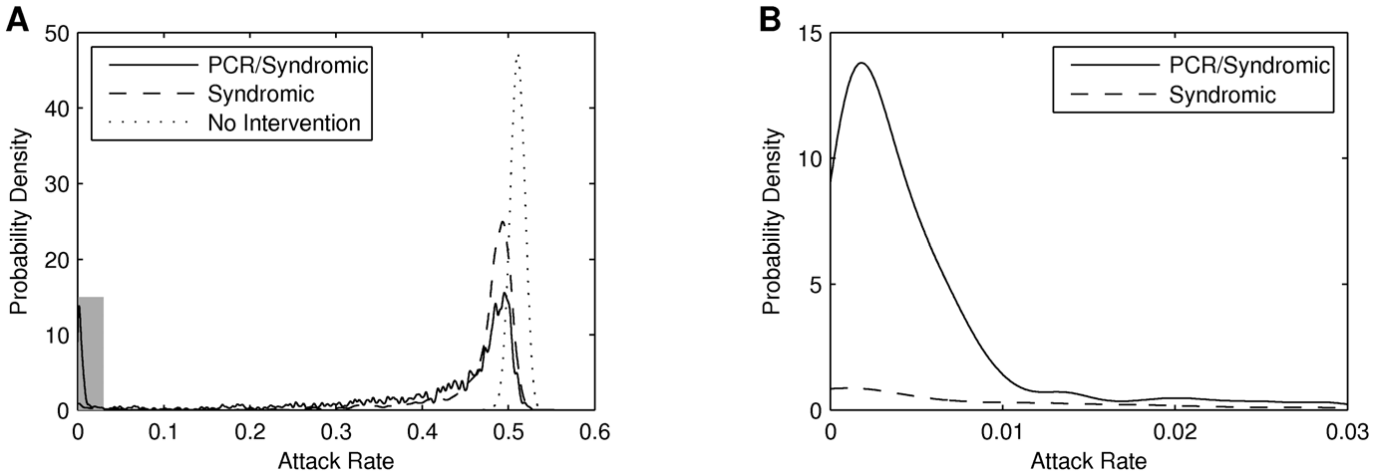
Probability densities of attack rate show the interventions have an “all or nothing” impact. **A:** Kernel smoothing density estimates for 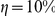 ( i. e. maximum severity). Both interventions shift the density leftwards, the expected attack rate now marginally less than without intervention. The PCR/Syndromic intervention shows a clear second peak at very low values for the attack rate. Few simulations result in an attack rate in the intervening space: the “all or nothing” impact. **B:** A magnified view of the shaded region in **A**, showing the second peak for the PCR/Syndromic strategy. The peak captures the simulations whereby antiviral distribution delayed the epidemic for sufficient time for the vaccine to be deployed and provide definitive control of the epidemic. It is noteworthy that almost all of the density under 10% (our and the AHMPPI 's working definition for successful mitigation) is in fact under 1%.

The validity of measuring impact as the proportion of simulations where the final attack rate was less than 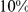 is demonstrated in Figure 4. Without an intervention the unmitigated epidemic results in a final attack rate of 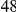
–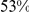; successful strategies such as syndromic diagnosis and PCRreduce the size of this peak and produce a long tail. Such strategies also exhibit a secondary peak for attack rates of 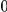
–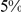, indicating an “all or nothing” effect. As we have previously shown [8], a finite stockpile of antivirals administered in isolation does little to reduce the final size of an epidemic, but may delay its peak. Scenarios in which the attack rate is reduced below the target threshold of 10% are those in which antivirals successfully buy time for vaccine implementation to control the outbreak. Across the different strategies, those with greater impact exhibit a smaller peak at high attack rates and a larger peak at low attack rates. This inverse relationship between the size of the two peaks demonstrates that our measure of impact is valid.

**Figure 5:**
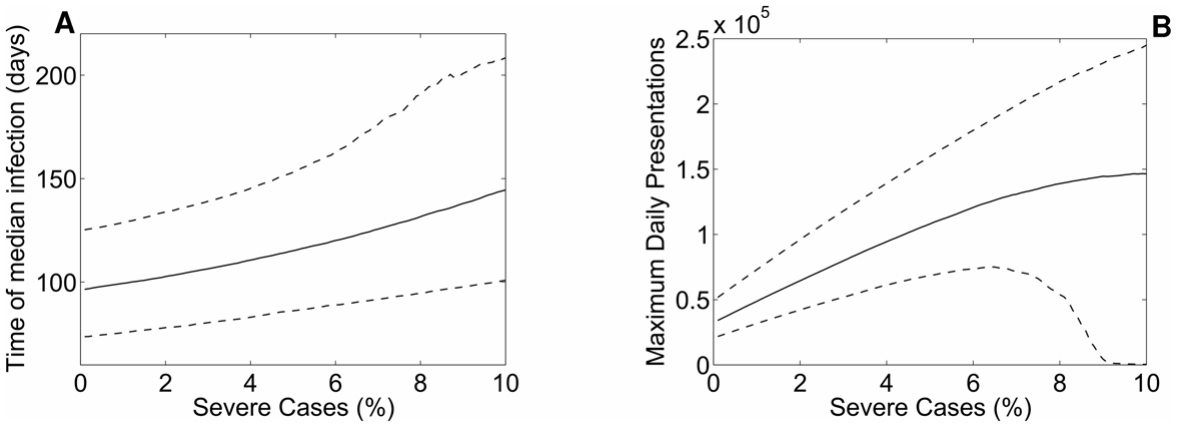
Timing and peak load of the epidemic as a function of the proportion of cases who are severe, under a PCR/Syndromic strategy. **A:** Time to median infection (50% of the final attack rate), in relation to influenza severity under the antiviral intervention. The solid line reports the median value from 2,000 simulations, dashed lines represent 5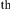 and 95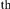 centiles. As severity (and hence the presenting proportion) increases, more cases are amenable to intervention, resulting in delays in epidemic growth of several weeks. At low severity the variation in model outputs is due to LHS parameter sampling. The upward trend in the 5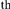, 50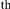 and 95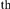 centiles with increasing severity shows the impact of the intervention to slow transmission, even where definitive control is not achieved. The increased scatter of observed values at high severity assumptions (characterised by increased upward trend in the 95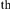 centile) reflects the ability of the vaccine to provide definitive control in a minority of simulations (see Figure 4). **B**: Maximum daily clinical presentations, in relation to influenza severity under the antiviral intervention. The solid line reports the median value from 2,000 simulations, dashed lines represent 5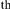 and 95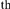 centiles. Presenting cases necessarily increase with epidemic severity given the model's assumptions. However, when severity is high (9–10%) the 5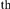 centile values collapse to approach 0, denoting the successfully “mitigated” (due to vaccination) epidemics.

Even where the epidemic is not successfully curtailed by immunisation, use of antivirals to constrain transmission may slow the rate of epidemic growth. Delays of several weeks to the time of median infection are observed when the severity (and hence the presenting proportion) is high (Figure 5A). Figure 5B reports the maximum number of clinical presentations per day in the context of the antiviral intervention. This figure is more complex to interpret, as symptomatic cases are expected to increase as a function of severity. It should be noted however, that as severity approaches 10%, the median number of presentations reported from the simulations begins to plateau. The declining median reflects successful containment by vaccination in a proportion of simulations, demonstrated by the downturn in the 5th centile values.

**Figure 6:**
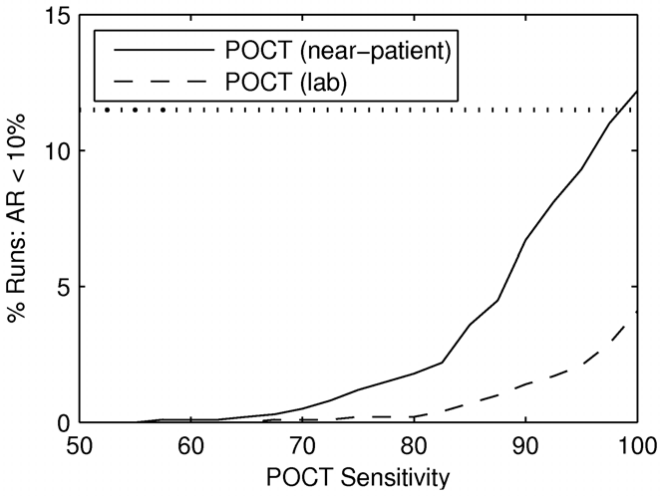
The impact of POCT s is hampered by low sensitivity. POCT s strategies are in principle capable of achieving equivalent results to PCR/Syndromic based strategies for antiviral deployment if sensitivity for the test is 100% and the outcome is assessed without delay (POCT (near-patient) for a sensitivity of 100%). The proportion of simulations in which control is achieved diminishes rapidly with falling sensitivity. Current sensitivity is around 60%. Transporting samples to external labs, thus introducing a delay from testing to provision of antiviral agents, further reduces the impact (POCT (lab)). The horizontal line marks the maximal impact of the PCR /Syndromic strategy.

The negligible impact of the POCT strategies is due to their relatively low sensitivity (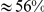 [27]). As shown in Figure 6, if we assume the POCT s are 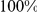 sensitive then the impact of the POCT near-patient strategy equals that of the PCRand PCR/syndromic strategies. However, as POCT sensitivity decreases, the impact is greatly reduced; impact is negligible when the sensitivity is 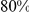.

The impact of analysing POCT s at external labs is much lower than the near-patient strategy; the optimal POCT impact (i. e. given 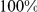 sensitivity) is decreased from 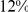 to 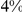. This three-fold reduction is due to the sole distinction between the two strategies: the effectiveness of treatment and prophylaxis delivered in response to GP presentations is decreased by 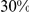 under the lab-based strategy. Since half of the mild presentations occur at GP s (on average), the reduced impact is equivalent to that of the near-patient POCT strategy with a sensitivity of only 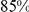, which highlights the importance of sensitive diagnosis and timely interventions.

In practice, the effective sensitivity of POCT s can be increased by performing cluster testing (e. g. across school classes or household units), but while cluster testing may be useful in identifying outbreaks in relatively closed communities (e. g. schools and events such as World Youth Day), it is unlikely to be of use in identifying the majority of cases once widespread community transmission is established. POCT sensitivity may also be higher in children than in adults [45]; since youth transmission initially sustained the epidemic in Victoria, Australia [46] and played an important role in the UK [41], the effective sensitivity of POCT s may well be higher than the estimate of 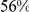 used here. However, even at these higher values (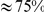) the impact would still have been minimal in comparison to the PCRand PCR/Syndromic strategies.

### Sensitivity to Delivery Constraints and Diagnostic Capacity

Given that the PCRand PCR/syndromic strategies have the greatest impact, it is instructive to analyse how the logistical constraints identified in this paper affect the impact of these two strategies. Recall that these diagnosis and antiviral delivery constraints limit the ability to diagnose presenting cases and to deliver prophylaxis to contacts of diagnosed cases, respectively. It is clear that these two constraints interact: given a low diagnosis capacity it is not possible to deliver a large amount of prophylaxis, regardless of the delivery capacity; conversely, given a low delivery capacity, additional diagnosis capacity will not produce an increased impact. Ideally these two constraints would be matched, with the diagnostic capacity capable of saturating—but not exceeding—the available delivery capacity.

**Figure 7:**
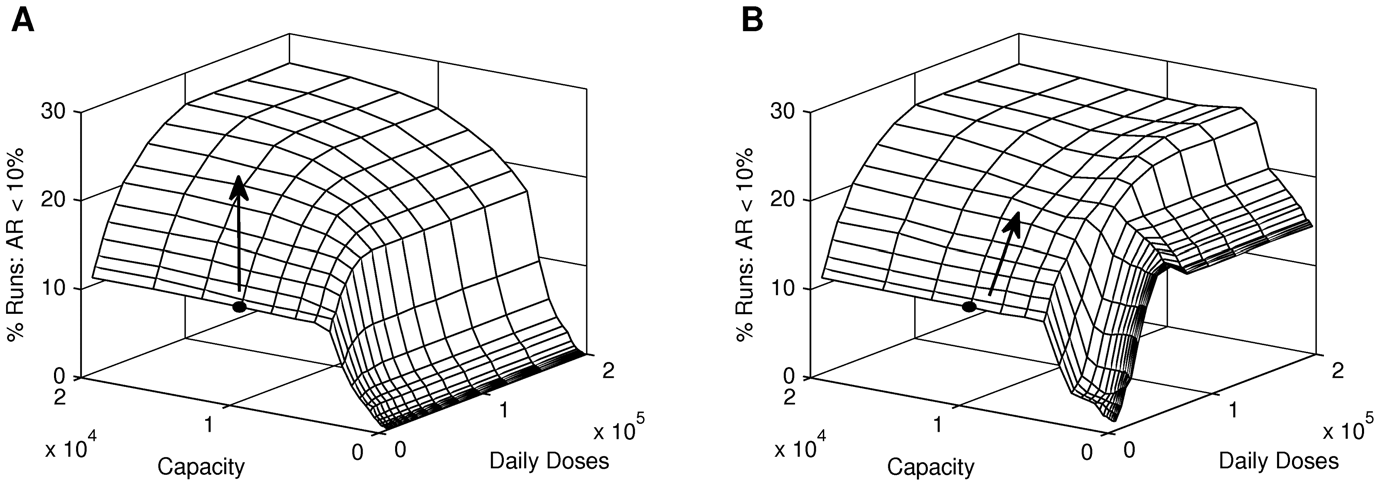
Impact of the PCR and PCR/Syndromic strategies, over a range of logistical constraints. **A:** Impact of using PCR tests. **B:** Impact when switching to syndromic diagnosis once PCR diagnostic capacity is exceeded. For both figures, the proportion of simulations that are effectively controlled increases non-linearly with both diagnostic (PCR) capacity and the maximum daily antiviral prophylaxis delivery capacity. Black circles indicate estimates of current Australian constraints; arrows indicate the direction in which to increase these resources to optimally increase the impact.

Figures 7A and 7B show the impact of the PCRand PCR/syndromic strategies, respectively, for a range of diagnostic and delivery capacities. Both strategies have an optimal impact of 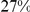, reinforcing the earlier result that these two strategies have similar impacts. However, when the diagnostic capacity is less than 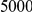 tests per day, the PCR/syndromic strategy has much greater impact than the PCRstrategy; this is because the diagnostic capacity is not sufficient for PCRtests to instigate maximal delivery of prophylaxis.

Under these conditions, the PCR/syndromic strategy has the greatest impact when prophylaxis delivery is capped at 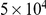 doses per day; when the delivery capacity is greater, the impact of this strategy is reduced by 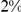 due to excessive delivery of prophylaxis to contacts of persons that are not infected with the pandemic strain. Once the diagnostic capacity is greater than 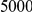 tests per day, syndromic diagnosis is delayed until the proportion of ILI presentations infected with the pandemic strain is sufficiently high (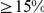) and delivery of prophylaxis to non-pandemic contacts does not reduce the PCR/syndromic impact.

As the diagnostic capacity is increased, both the PCRand PCR/syndromic strategies approach maximal impact (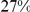); a capacity of 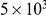 is sufficient to achieve maximal impact using the PCR/syndromic strategy, while a capacity of 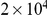 is needed to achieve maximal impact using the PCRstrategy. This four-fold difference in diagnostic capacities demonstrates that once a high proportion of ILI presentations are infected with the pandemic strain, syndromic diagnosis is more effective than using PCRtests, within the logistical constraints identified here.

From this analysis, one may infer that the optimal combination of capacities is such that the diagnostic capacity is sufficient to saturate—but not overload—the prophylaxis delivery capacity. Assuming that incidence of the pandemic strain peaks at 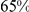 of ILI, the optimal strategy is to continue with PCRtests until the available lab capacity is only 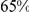 of the daily number of samples, then switching to syndromic diagnosis. Such a strategy requires instant surveillance data and that PCRlab capacity is devoted solely to testing the samples delivered that day, ignoring any backlog of samples from previous days—both assumptions are highly unrealistic. Furthermore, a few days (at most) would elapse between the saturation of PCRlab capacity and this optimal switching time. Meanwhile, the combined PCR/syndromic strategy (Figure 7B) has very similar impact and is also realistically achievable.

This model assumes a homogeneous population and uniform distribution of diagnostic capacity, whereas an actual epidemic will be inhomogeneous across the country [47]. Thus, the recommended course of action is for each locality (i. e. state) to use PCRtests as the decision making tool until the locally available lab capacity is exceeded, at which point the presentation of ILI should be used as the decision making tool.

Figures 7A and 7B show the impact of the PCRand PCR/syndromic strategies, where the grey circles indicate our estimates of the current Australian healthcare system resources; in both cases, the current constraints are decidedly sub-optimal. On each figure, arrows indicate alternate courses for increasing these resources and it is apparent that the most effective course differs depending on which diagnosis strategy is used. Increasing the maximal prophylaxis delivery rate produces the largest increase in impact for either strategy; for the PCRstrategy a further (small) increase can then be produced by doubling the available lab capacity. In both cases, the impact is optimal when the maximal delivery of prophylaxis is increased *ten-fold*, to 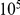 doses per day.

## Discussion

### Key findings

The impact of any combination of diagnosis strategies and antiviral intervention on an influenza epidemic depends on the proportion of infections that present, the inherent properties of the diagnosis strategy and antiviral intervention, and the constraints placed upon the intervention by limited healthcare resources. The key attributes of a successful diagnostic strategy were shown to be a large diagnostic capacity and very high sensitivity. In the early stages of an epidemic—when the proportion of ILI presentations infected with the pandemic strain is negligible—it is also important that the strategy is highly specific, to make optimal use of the limited antiviral distribution capacity and to avoid early depletion of the antiviral stockpile.

The optimal strategy for targeting antiviral interventions was a combination of PCRtests early in the epidemic, and syndromic diagnosis once the PCRlab capacity was exceeded; this strategy is estimated to have a 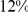 chance of mitigating an extremely severe epidemic. Because of the ability to switch to syndromic diagnosis, the results suggest that directing additional resources to increasing laboratory diagnostic throughput will have negligible influence on the impact of a strategy of mass antiviral prophylaxis.

Using PCRtests as the sole diagnostic tool resulted in a similar impact, under the unlikely assumption that the lab capacity was devoted solely to testing newly-arrived samples and that any backlog was ignored. The use of syndromic diagnosis from the outset was shown to have less impact—a 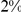 chance of mitigation—due to very low specificity in the early stages of the epidemic, while POCT s were shown have no impact, due to low sensitivity. The sensitivity of POCT s could be improved by performing cluster testing, an option that was not explored here.

Based on the estimates of the current logistical constraints of the healthcare system, a sensitivity analysis was conducted to determine how the impact of the most successful strategies were affected by the available diagnostic and delivery capacities. The estimated Australian PCRdiagnostic capacity of 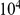 tests per day was shown to be optimal for the PCR/syndromic strategy and near-optimal for the PCRstrategy. In contrast, the maximal rate of prophylaxis delivery was estimated to be an order of magnitude less than the optimal rate of 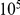 doses per day, with significant implications for epidemic mitigation. These findings suggest that optimal allocation of additional resources to build capacity should be directed towards drug delivery rather than laboratory testing.

### Strengths and weaknesses

We have made a number of simplifying assumptions to ensure that our model is tractable. Population heterogeneity and clustering [47] is ignored, and asymptomatic and symptomatic cases have identical infectiousness. While the model does not consider the influence of emergent resistance to antiviral agents on intervention effectiveness, modelling studies that account for resistance suggest that widespread antiviral deployment would remain an effective mitigation strategy [9], [48]–[55].

We assume that diagnosis at flu clinics is sufficiently rapid to deliver timely treatment to patients and timely prophylaxis to contacts, that the available hospital capacity can cater for all severe cases, and only consider those cases as “presenting” who attend medical services in a timely manner. At the beginning of the epidemic the whole population is susceptible (i. e. immunologically naive). The model is also non-stochastic (deterministic), which can be problematic when 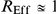.

The strength of this model lies in the ability to account for pragmatic issues; while previous modelling studies have aimed to identify optimal vaccine distribution strategies (e. g. [56], [57]), predicting the effects of diagnosis and distribution capacities on the impact of antiviral interventions is a novel application of SEIR models, and the results highlight the importance of specific planning to develop feasible and effective healthcare responses.

Significantly, by taking into account logistical constraints that were observed in pandemic responses world-wide, our results suggest the increasing lab diagnostic capacity may have little or no effect on the impact of a pandemic response.

### Implications for healthcare policy

The optimal antiviral targeting strategy identified here is to use PCRtests to diagnose pandemic cases until the available lab capacity is exceeded, from which point syndromic diagnosis (the presence of ILI symptoms) should be used. Solely using PCRtests for the duration of the epidemic can produce a similar impact when priority is given to the most recently received samples, but this strategy is more resource-intensive and would place great stress on the labs and on the couriers transporting samples to the labs; the last-in first-served test analysis is also unlikely to be realised, due to practical considerations such as the role that labs play in surveillance.

Given our estimates of the current capacity constraints of the healthcare system, the optimal strategies have a 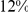 chance of mitigating an epidemic (under the scenarios described in the Methods section) when the severity is highest (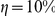), since this drives the greatest proportion of mild cases to present. Contrary to expectations, a sensitivity analysis of these strategies showed that the PCRdiagnostic capacity is optimal and that the ability to deliver large amounts of prophylaxis on a daily basis is the key constraint. This suggests that capacity building resources would be better committed to developing creative approaches to decentralised contact identification and delivery, rather than increasing lab diagnostic capacity. Compared to our estimated rate of 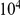 doses per day, the optimal rate is 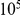 doses per day, which more than doubles the chance of mitigating an epidemic to 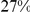. An added advantage of adopting a decentralized approach is the ability to reduce peak workload on specialized public health response teams, reducing burnout and ensuring ongoing capability to respond to evolving priorities as the epidemic unfolds.

Achieving this delivery rate represents a serious challenge for the healthcare sector. Notwithstanding ethical and legal complications, this is not an insurmountable goal; Australia Post delivers around 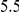 *billion* articles per year (i. e. 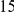 million *per day*) to almost 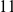 million addresses inside Australia, with 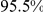 of articles being delivered on time and 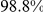 of delivery points being serviced 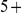 days per week [58]. The Mail & Networks division of Australia Post has 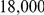 full-time employees, 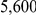 part-time employees and 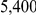 “other” employees, for a total of 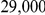 people [58]; to deliver 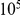 doses of prophylaxis per day, a workforce of 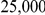 people across the nation could be tasked with delivering prophylaxis to four persons every day. This demonstrates that a decentralised delivery infrastructure could deliver the necessary number of prophylaxis doses, assuming that solutions can be found for the associated ethical and legal issues. Adoption of this “whole of society” strategy further offloads pressure on the health sector, which has sole capability to deliver other essential acute care services that will be operating at full surge capacity during a raging epidemic.

Creative solutions were employed during the 2009 pandemic response locally and internationally, to facilitate prescribing and distribution of antiviral agents. In the state of Victoria in Australia, couriers were initially used to distribute prophylactic antiviral agents prescribed through the Department of Human Services, a role that could similarly have been fulfilled by Australia Post (Dr Rosemary Lester, personal communication). Within weeks of the outbreak's commencement, Division 1 nurses were given the right to prescribe antiviral drugs for prophylaxis without medical consultation [59]. Building on the existing capacity of telephone consultation services provided through the NHS, the United Kingdom implemented a National Pandemic Flu Service for self-care advice via internet or telephone to reduce the pressure on primary care and General Practitioners [60].

The maximal impact of the strategies considered here is a 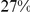 chance to mitigate an epidemic, given a 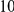-fold increase in prophylaxis delivery and a mild presentation proportion of 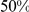. This low impact (a 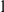-in-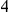 chance) indicates the importance of effective social interventions (such as school closures and the cancellation of public events) to reduce exposure to the pandemic strain [61]. Evidence from Japan is suggestive that widespread use of antivirals and widespread school closures were highly effective in 2009 [62] and school closures were also found to be effective in Hong Kong in 2009 [63]. The ability of such measures to reduce the effective reproduction rate can greatly increase the impact of the strategies presented here.

Furthermore, when the proportion of infected persons that present is low, antiviral interventions will have minimal impact at best and there is little to distinguish between the available diagnosis strategies. To illustrate this point, the 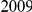 pandemic was milder than expected, with a low proportion of presentations; accordingly, none of the strategies described here are likely to have had any impact on the epidemic.

### Summary

We address specific real-world issues that must be considered in order to improve pandemic preparedness policy in a practical and methodologically sound way. Consistent with expectations, we identify the importance of sensitive and specific case ascertainment in the early phase of a pandemic response, when the proportion of false-positive presentations may be predicted to be high. However, once a substantial percentage of ILI presentations are caused by the pandemic strain, identification of cases (and contacts) to be treated on syndromic grounds alone results in a more streamlined response that has greater potential to be effective than a laboratory-dependent strategy. Beyond this threshold, there is little benefit for the outcome of the antiviral intervention in increasing laboratory diagnostic capacity—rather, our model's findings reinforce the need for a decentralised drug delivery system capable of providing prophylaxis to contacts in a timely manner.

Provision of antivirals on the scale proposed for an effective response is infeasible using traditional public health outbreak management and contact tracing approaches. The results indicate that for an antiviral intervention to change the transmission dynamics of an influenza epidemic, a decentralised system is required for contact identification and prophylaxis delivery, utilising a range of existing services and infrastructure in a “whole of society” response.

Whilst some countries have considered utilising decentralised infrastructures, centralised systems remain the dominant platform for pandemic response strategies. In addition, reviews of pandemic preparedness have recommended greatly increasing laboratory diagnostic capacity [20], while the logistics of prophylaxis distribution have received less attention. Our results present a challenge to this status quo.

## Supporting Information

Supplementary Material S1 We provide the details of the model, including all of the model equations, in Section S1. The model parameters are then described in detail, including the probability distributions used for the Latin hypercube sampling (LHS), in Section S2.
(0.17 MB PDF)
