# Supplementary material for: Diagnosis and Antiviral Intervention Strategies for Mitigating an Influenza Epidemic
Source: PLoS One. 2011 Feb 4;6(2):e14505. doi: 10.1371/journal.pone.0014505 (PMC3033893; doi:10.1371/journal.pone.0014505)
Supplement: Supplementary Material S1 — We provide the details of the model, including all of the model equations, in Section S1. The model parameters are then described in detail, including the probability distributions used for the Latin hypercube sampling (LHS), in Section S2. (0.17 MB PDF) [file pone.0014505.s001.pdf]

Supplementary Material

We provide the details of the model, including all of the model equations, in Section S1. The model parameters are then described in detail, including the probability distributions used for the Latin hypercube sampling (LHS), in Section S2.

S1 Detailed model structure

The model used in this paper involves several modifications to the contact model first introduced in [1] and further developed in [2]. Here we describe these modifications: changes to model states and parameters (Section S1.1); case presentation, diagnosis and antiviral deployment (Section S1.2); and the effect of vaccination on infection (Section S1.3). The model structure is shown in Figure 1.

S1.1 Changed definitions and new parameters

The model presented here assumes that asymptomatic and symptomatic individuals are equally infectious (this corresponds to $\chi = 1$ in the original model [2]), rendering the distinction between symptomatic and asymptomatic infections redundant. Accordingly, the I and A states distinguish between *presenting* and *non-presenting* cases, unlike [2] where they distinguished between symptomatic and asymptomatic infections.

The parameter α is the proportion of cases that present (to hospitals or to outpatient facilities). The basic reproduction number (R_0) of the pandemic influenza strain and the inverse infectious period (γ) are explicit parameters of this model; the number of infections per unit time made by an infectious individual (β) is not.

In recognition that not all contacts of an infectious individual can be identified and provided with post-exposure prophylaxis, we introduce a parameter σ that defines the proportion of contacts that are potentially identifiable. Accordingly, the proportion of all contacts that receive prophylaxis (ϵ) cannot exceed σ.

Finally, the fraction of presenting cases that receive treatment (ψ) and the fraction of contacts that receive prophylaxis (ϵ) are functions of time, since they are both affected by the logistical constraints introduced in this model and do not remain constant throughout an epidemic.

S1.2 Presentation, diagnosis and antiviral deployment

The proportion of all infected cases that present (α) is the sum of the severe cases (all of which present) and the proportion (α_M) of the remaining (i. e. mild) cases that present:

$$\alpha = \eta + \alpha_M(1 - \eta) \quad \text{(S1)}$$

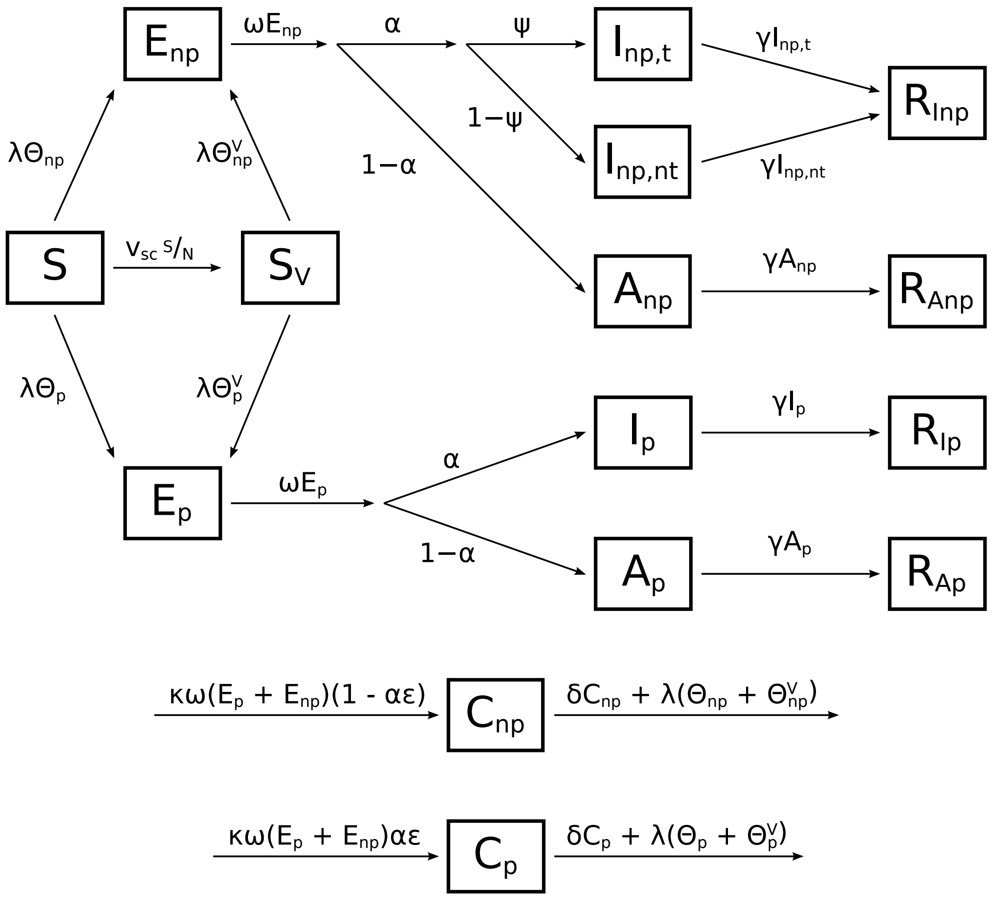

Figure 1: The flow between the state variables in the model where ψ and ϵ are functions of time, unlike in [2]. The contact classes C_{np} and C_p are labels for tracking contact status and are orthogonal to the SEIR states; see [1] for further details.

We have assumed that α_M is dependent on the severity of the epidemic (η); the probability distribution for α_M is a function of η and is presented in Section S2.2.

All severe cases present at hospitals and receive timely diagnosis and treatment; only mild cases (i. e. outpatient presentations) are subject to constraints on diagnosis and treatment. The rate at which mild cases present is denoted $\dot{P_M}$:

$$\begin{aligned}\dot{P_M} &= \alpha\omega(E_p + E_{np}) \times \frac{\alpha_M(1-\eta)}{\eta + \alpha_M(1-\eta)} \\ &= [\alpha_M(1-\eta)]\omega(E_p + E_{np})\end{aligned} \tag{S2}$$

Given the rate of mild presentations and an estimate of the proportion of influenza-like illness (ILI) presentations that are infected with the pandemic strain ($\text{ILI}_{\%}$), the rate of ILI presentations ($\dot{P_{ILI}}$) can be calculated:

$$\dot{P_{ILI}} = \frac{\dot{P_M}}{\text{ILI}_{\%}(t)} \tag{S3}$$

The rate at which positive diagnoses for pandemic influenza ($\dot{D_P}$) are returned from outpatient ILI presentations is the sum of the true positives (i. e. pandemic cases) and the false positives, subject to a maximal diagnosis rate of MAX_D. The parameters s_N and s_P are the fraction of true positives and true negatives that are identified across all outpatient ILI presentations, respectively.

$$\dot{D_P} = \min\left(\text{MAX}_D, \dot{P_{ILI}}\right) \times \left(\text{ILI}_{\%}(t)s_N + (1 - \text{ILI}_{\%}(t))(1 - s_P)\right) \tag{S4}$$

The rate of true positives is given by $\dot{D_{TP}}$:

$$\dot{D_{TP}} = \min\left(\text{MAX}_D, \dot{P_{ILI}}\right) \times \text{ILI}_{\%}(t)s_N \tag{S5}$$

Antivirals are deployed as treatment only to positively-diagnosed ILI presentations who did not receive antivirals for prophylaxis, subject to a maximal delivery rate of MAX_T:

$$\dot{AV_T} = \min\left(\text{MAX}_T, \frac{E_{np}}{E_p + E_{np}}\dot{D_P}\right) \tag{S6}$$

The rate at which *effective* treatment is delivered to mild cases ($\dot{T_M}$) is the fraction of the antiviral deployment that is delivered to pandemic cases, where the efficacy of antivirals delivered as a result of general practice (GP) presentations (f_{GP}) is reduced by e_{GP} to account for delays inherent in analysing samples at external labs:

$$\dot{T_M} = \dot{AV_T} \frac{\dot{D_{TP}}}{\dot{D_P}} \times \Big(e_{GP} f_{GP} + (1 - f_{GP}) \Big) \tag{S7}$$

The fraction of all presenting cases that receive treatment (ψ) is the sum of the mild cases that receive effective treatment and the severe cases (all of which receive effective treatment):

$$\psi(t) = \frac{\dot{T_M} + \eta\omega(E_p + E_{np})}{\alpha\omega(E_p + E_{np})} \tag{S8}$$

Antivirals are deployed as prophylaxis to a fraction σ of the contacts of all severe cases and of all ILI presentations that return a positive diagnosis ($\dot{D_P}$), subject to a maximal delivery rate of MAX_P:

$$\dot{AV_P} = \min \Big(\mathrm{MAX}_P, \kappa\sigma \Big(\dot{D_P} + \eta\omega(E_p + E_{np}) \Big) \Big) \tag{S9}$$

The rate at which prophylaxis is delivered to contacts of pandemic cases ($\dot{P_{TP}}$) is the fraction of the antiviral deployment that is delivered to contacts of pandemic cases. We approximate this fraction to be $\frac{\dot{D_{TP}}}{\dot{D_P}}$, which discounts the fact that all severe cases are correctly diagnosed; the justification for this approximation is that mild cases represent the bulk of the pandemic infections and are the key to controlling transmission in the community. As per the delivery of effective treatment, the efficacy of antivirals delivered as a result of GP presentations is reduced by e_{GP} to account for delays inherent in analysing samples at external labs:

$$\dot{P_{TP}} = \dot{AV_P} \frac{\dot{D_{TP}}}{\dot{D_P}} \times \left(\frac{\eta}{\alpha} + \frac{(1-\eta)\alpha_M}{\alpha} [e_{GP} f_{GP} + (1 - f_{GP})] \right) \tag{S10}$$

The fraction of all contacts that receive prophylaxis (ϵ) is the rate at which prophylaxis is delivered to these contacts, divided by the total number of contacts:

$$\epsilon(t) = \frac{\dot{P_{TP}}}{\kappa\alpha\omega(E_p + E_{np})} \tag{S11}$$

Finally, the antiviral stockpile is depleted due to the *total* number of antiviral doses distributed for treatment and for prophylaxis:

$$\frac{dO}{dt} = -\dot{AV_T} - \dot{AV_P} \tag{S12}$$

S1.3 Vaccine distribution and infection

The original SEIR model [2] introduced Θ_p and Θ_{np}, which define the proportion of susceptible contacts in the population:

$$\Theta_p = \frac{e_s C_p}{C_P + C_{np}} \times \frac{S}{N} \tag{S13}$$

$$\Theta_{np} = \frac{C_{np}}{C_P + C_{np}} \times \frac{S}{N} \tag{S14}$$

Here we introduce similar variables Θ_p^V and Θ_{np}^V, which define the proportion of susceptible *vaccinated* contacts, who have a reduced susceptibility e_v due to successful seroconversion:

$$\Theta_p^V = \frac{e_s C_p}{C_P + C_{np}} \times \frac{e_v S_V}{N} \tag{S15}$$

$$\Theta_{np}^V = \frac{C_{np}}{C_P + C_{np}} \times \frac{e_v S_V}{N} \tag{S16}$$

The force of infection (λ) arises from the five infectious classes just as in the original SEIR model [2], given the number of infections per unit time made by an infectious individual (β):

$$\beta = R_0 \times \gamma \tag{S17}$$

$$\lambda_p = \beta e_i (I_p + A_p) \tag{S18}$$

$$\lambda_{np,nt} = \beta (I_{np,nt} + A_{np}) \tag{S19}$$

$$\lambda_{np,t} = \beta e_t I_{np,t} \tag{S20}$$

$$\lambda = \lambda_p + \lambda_{np,nt} + \lambda_{np,t} \tag{S21}$$

We introduce a new state S_V for vaccinated susceptibles (shown in Figure 1). People move from S to S_V in proportion to the rate of seroconversion v_{SC}:

$$\frac{dS}{dt} = -\lambda(\Theta_P + \Theta_{np}) - v_{SC} \times \frac{S}{N} \tag{S22}$$

$$\frac{dS_V}{dt} = -\lambda(\Theta_P^V + \Theta_{np}^V) + v_{SC} \times \frac{S}{N} \tag{S23}$$

The vaccine seroconversion rate v_{SC} is held at zero until week 20 of the epidemic, under the assumption that a vaccine becomes available 18 weeks into the epidemic and that seroconversion occurs two weeks after receiving a single dose of vaccine [3].

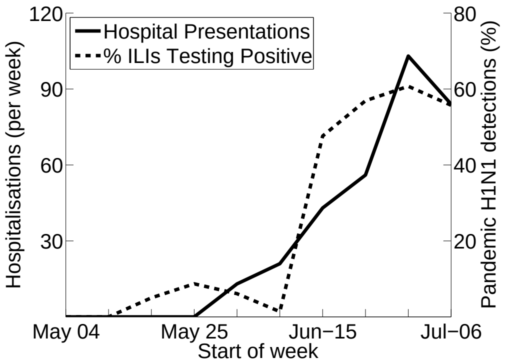

Figure 2: The relationship between the proportion of ILI cases that test positive for pandemic influenza and the number of pandemic hospitalisations per week.

S2 Model parameters

The model parameters are now presented in detail. We begin with an estimate of the proportion of ILI presentations infected with pandemic influenza at any point in an epidemic. This is followed by a discussion of the basic reproduction number and how it compares to estimates from the 2009 pandemic. Finally, we provide the probability distributions that were used by the Latin hypercube sampling (LHS) algorithm to select parameter values.

S2.1 Estimating ILI presentations from pandemic presentations

Assuming a finite diagnosis capacity, the number of pandemic cases that are diagnosed depends on the proportion of ILI presentations that are infected with the pandemic strain ($\text{ILI}_{\%}$). Victorian surveillance data from the 2009 epidemic indicates that this proportion is almost 0% early in the epidemic, raising to as high as 65% at the epidemic peak [4, 5]. Combining surveillance data with hospitalisation data [6]—shown in Figure 2—permits a linear model relating $\text{ILI}_{\%}$ to pandemic presentations to be fitted, assuming that the hospitalised cases represent a fixed proportion of pandemic presentations (we assumed 0.5% to fit the linear model presented here). We add the constraint $\text{ILI}_{\%} \in [3\%, 65\%]$ to avoid stiffness issues with the MATLAB ODE solver, and arrive at the following relationship:

$$\text{ILI}_{\%}(t) = \alpha\omega(E_p + E_{np}) \times \frac{3 \times 10^{-5}}{52} \qquad (0.03 \leq \text{ILI}_{\%} \leq 0.65) \tag{S24}$$

S2.2 Probability distributions for model parameters

The model parameters can be divided into two categories: those that are independent of the chosen diagnosis strategy and those that are specific to the chosen di-

Parameter	A	$A + B \times \mathrm{Beta}(\mu, V)$<br>$B$	μ	V	Description
R_0	1.35	0.1	0.5	0.1	Basic reproduction number
ω	180	550	0.3364	0.1636	Inverse latent period (years^{-1})
γ	146	219	0.4444	0.2283	Inverse infectious period (years^{-1})
δ	121.6667	60.8333	0.4	0.1973	Inverse contact period (years^{-1})
κ	20	20	0.5	0.25	Average number of contacts (Contain phase)
κ	12	16	0.5	0.25	Average number of contacts (Sustain phase)
e_t	0.8	0.2	0.9	0.04	Infectiousness due to treatment
e_s	0.2	0.6	0.5	0.1667	Susceptibility due to prophylaxis
e_i	0.8	0.2	0.9	0.04	Infectiousness of breakthrough cases
α_m		see Figure 3			Proportion of mild cases that present

Parameter	$\mathrm{Uni}(X_{\min}, X_{\max})$<br>$X_{\min}$	$X_{\max}$	Description
η	0.1%	10%	Proportion of severe cases
f_{GP}	0.2	0.8	Proportion of outpatient presentations at GPs

Parameter	Value	Description
N	20×10^6	Population size (people)
O	8×10^6	Antiviral stockpile size (doses)
MAX_T	1×10^4	Maximal treatment rate (doses per day)
MAX_P	1×10^4	Maximal prophylaxis rate (doses per day)
v_{SC}	5.25×10^5	Rate of vaccine seroconversions (persons per week)
e_v	0.3	Susceptibility due to successful vaccination
σ	0.5	Proportion of contacts that are feasibly traceable
s_N	see Table 2	ILI presentations diagnosed as true positives
s_P	see Table 2	ILI presentations diagnosed as true negatives
MAX_D	see Table 2	Maximum number of outpatient diagnoses (per day)
e_{GP}	see Table 2	Antiviral efficacy for GP-diagnosed cases and contacts

Table 1: Probability distributions for the model parameter; each parameter is associated with a beta distribution, a uniform distribution, or a single value.

agnosis strategy. The probability distributions for the strategy-independent parameters are listed in Table 1, while the values of the strategy-specific parameters are listed in Table 2. The probability distribution for α_M is a function of η and is shown in Figure 3.

S2.3 Basic reproduction number

Our value of $R_0 \approx 1.4$ is smaller than a conservative estimate for the early growth phase of the 2009 Victorian epidemic, where $R_0 \approx 1.6$ when correcting for undetected transmission, but the same study found that $R_0 < 1$ except for youth-to-youth transmissions [7]. Our R_0 value is consistent with whole-wave estimates from the UK [8] and there is consistent serological evidence to suggest that the observed attack rates were low due to significant amounts of preexisting immunity [9]. Here we have assumed that the entire population is initially susceptible, which explains why our model produces more severe epidemics with $R_0 \approx 1.4$ than what was observed in 2009.

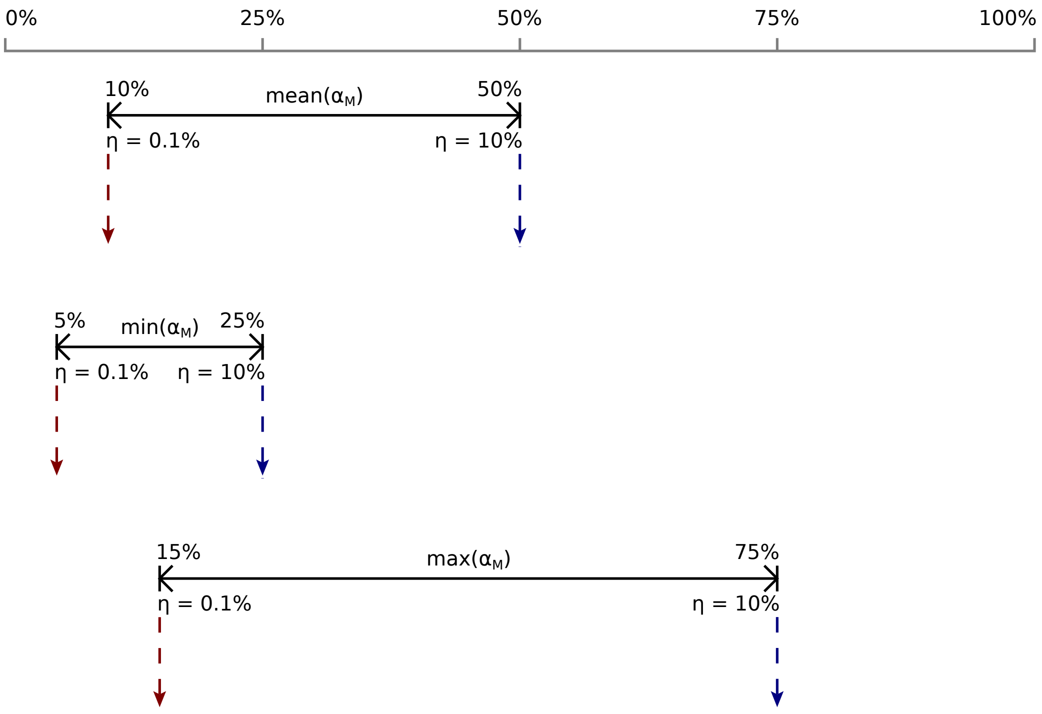

(a) The mean, minimum and maximum values for α_m are linear functions of η ($\eta \in [0.1\%, 10\%]$).

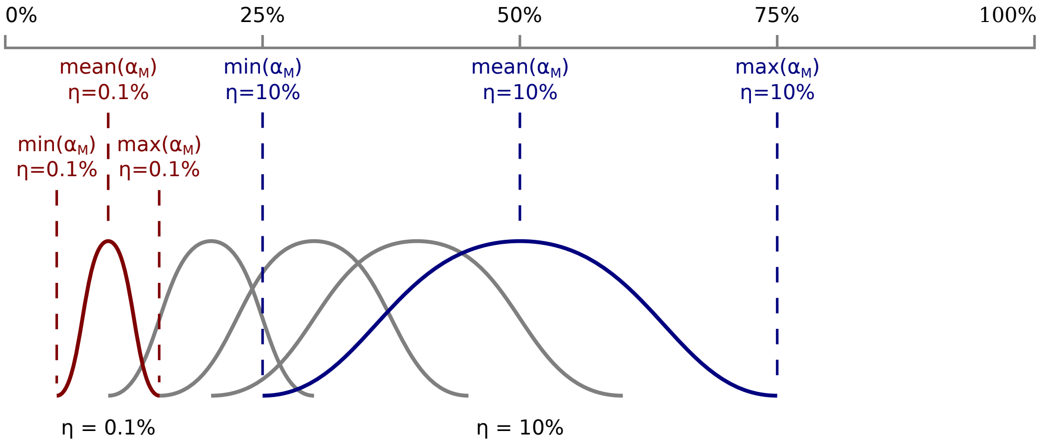

(b) The probability distribution for α_m when $\eta = 0.1\%$, 2.575%, 5.05%, 7.525% and 10%; the distributions for $\eta = 0.1\%$ and $\eta = 10\%$ are shown in red and blue. This distribution is given by: $\min(\alpha_m) + [\max(\alpha_m) - \min(\alpha_m)] \times \text{Beta}(\mu = 0.5, V = 0.2)$.

Figure 3: The probability distribution for mild presentations (α_M).

Strategy	s_N	s_P	MAX_D	e_{GP}
polymerase chain reaction (PCR)	1.0	1.0	10^4	0.7
Syndromic	1.0	0.0	N	1.0
point-of-care test (POCT) (near-patient)	0.56	0.9	N	1.0
POCT (lab)	0.56	0.9	10^5	0.7

Table 2: Values for parameters specific to a diagnosis strategy; $\mathrm{MAX}_D = N$ reflects a capacity sufficient to diagnose all outpatient ILI presentations.

References

[1] James M McCaw and Jodie McVernon. Prophylaxis or treatment? Optimal use of an antiviral stockpile during an influenza pandemic. *Math Biosci*, 209(2):336–360, Oct 2007, `doi:10.1016/j.mbs.2007.02.003`. URL `http://dx.doi.org/10.1016/j.mbs.2007.02.003`.

[2] Jodie McVernon, James M. McCaw, and Terence M. Nolan. Modelling strategic use of the national antiviral stockpile during the CONTAIN and SUSTAIN phases of an Australian pandemic influenza response. *Aust NZ J Publ Health*, 34(2): 113–119, 2010, `doi:10.1111/j.1753-6405.2010.00493.x`.

[3] Michael E Greenberg, Michael H Lai, Gunter F Hartel, Christine H Wichems, Charmaine Gittleson, Jillian Bennet, Gail Dawson, Wilson Hu, Connie Leggio, Diane Washington, and Russell L Basser. Response after one dose of a monovalent influenza A (H1N1) 2009 vaccine – preliminary report. *N Engl J Med*, Sep 2009, `doi:10.1056/NEJMoa0907413`. URL `http://dx.doi.org/10.1056/NEJMoa0907413`.

[4] Heath A Kelly, Kristina A Grant, Simon Williams, James Fielding, and David Smith. Epidemiological characteristics of pandemic influenza H1N1 2009 and seasonal influenza infection. *Med J Aust*, 191(3):146–149, Aug 2009.

[5] H. Kelly and K. Grant. Interim analysis of pandemic influenza (H1N1) 2009 in Australia: surveillance trends, age of infection and effectiveness of seasonal vaccination. *Euro Surveill*, 14(31), Aug 2009.

[6] M. E. Lum, A. J. McMillan, C. W. Brook, R. Lester, and L. S. Piers. Impact of pandemic (H1N1) 2009 influenza on critical care capacity in Victoria. *Med J Aust*, 191(9):502–506, 2009.

[7] E. S. McBryde, I. Bergeri, C. van Gemert, J. Rotty, E. J. Headley, K. Simpson, R. A. Lester, M. Hellard, and J. E. Fielding. Early transmission characteristics of influenza A(H1N1)v in Australia: Victorian state, 16 May - 3 June 2009. *Euro Surveill*, 14(42), 2009.

[8] Christophe Fraser, Christl A Donnelly, Simon Cauchemez, William P Hanage, Maria D Van Kerkhove, T. Déirdre Hollingsworth, Jamie Griffin, Rebecca F

Baggaley, Helen E Jenkins, Emily J Lyons, Thibaut Jombart, Wes R Hinsley, Nicholas C Grassly, Francois Balloux, Azra C Ghani, Neil M Ferguson, Andrew Rambaut, Oliver G Pybus, Hugo Lopez-Gatell, Celia M Apluche-Aranda, Ietza Bojorquez Chapela, Ethel Palacios Zavala, Dulce Ma Espejo Guevara, Francesco Checchi, Erika Garcia, Stephane Hugonnet, Cathy Roth, and The WHO Rapid Pandemic Assessment Collaboration. Pandemic potential of a strain of Influenza A (H1N1): Early findings. *Science*, 324(5934):1557–1561, May 2009, doi:10.1126/science.1176062.

[9] Elizabeth Miller, Katja Hoschler, Pia Hardelid, Elaine Stanford, Nick Andrews, and Maria Zambon. Incidence of 2009 pandemic influenza A H1N1 infection in England: a cross-sectional serological study. *Lancet*, Jan 2010, doi:10.1016/S0140-6736(09)62126-7. URL http://dx.doi.org/10.1016/S0140-6736(09)62126-7.
